# Active bleeding from a periampullary duodenal diverticulum that was difficult to diagnose but successfully treated using hemostatic forceps: a case report

**DOI:** 10.1186/1752-1947-6-367

**Published:** 2012-10-26

**Authors:** Noriko Nishiyama, Hirohito Mori, Kazi Rafiq, Hideki Kobara, Shintarou Fujihara, Mitsuyoshi Kobayashi, Tsutomu Masaki

**Affiliations:** 1 Departments of Gastroenterology and Neurology, Faculty of Medicine, Kagawa University, 1750-1 Miki, Kagawa, Kita, 761-0793, Japan; 2 Department of Pharmacology, Faculty of Medicine, Kagawa University, 1750-1 Miki, Kagawa, Kita, 761-0793, Japan

**Keywords:** Bleeding periampullary duodenal diverticulum, Side-viewing endoscopy, Hemostatic forceps, Obscure gastrointestinal bleeding

## Abstract

**Introduction:**

Although duodenal diverticula are common, periampullary duodenal diverticula are rare. Periampullary duodenal diverticula are usually asymptomatic and may be difficult to diagnose and treat. However, they may present with massive bleeding, requiring prompt diagnosis.

**Case presentation:**

We report the case of a 71-year-old Asian woman with bleeding from a periampullary duodenal diverticulum. She presented with severe anemia and tarry stools. Two examinations using a forward-viewing endoscope did not identify the source of the bleeding. However, examination using a side-viewing endoscope found an exposed bleeding vessel overlying the bile duct within a periampullary diverticulum of the descending part of the duodenum. The bleeding was successfully controlled by using hemostatic forceps.

**Conclusions:**

Bleeding periampullary duodenal diverticula are rare, and a bleeding point in the mucosa overlying the bile duct within a large periampullary duodenal diverticulum is very rare. Identification of a bleeding point within a duodenal diverticulum often requires repeated examination and may require the use of a side-viewing endoscope. Use of hemostatic forceps to control bleeding from a periampullary duodenal diverticulum is very rare but, for bleeding lesions overlying the bile duct within a periampullary duodenal diverticulum, is the best way to prevent obstructive jaundice.

## Introduction

The reported prevalence of duodenal diverticula in patients undergoing diagnostic examinations varies from 0.16% to 22% [1]. Duodenal diverticula are usually asymptomatic and are investigated only when symptomatic [2]. Periampullary duodenal diverticula are rare, and only a few cases have been reported to date. Their location makes it difficult to identify a bleeding point within the diverticulum, and severe hemorrhage from such a lesion is often life-threatening. Various therapeutic modalities, such as embolization, surgery, and advanced endoscopic therapy such as hemoclip, coagulation, or injection, may be used to control the bleeding [3,4]. However, there is no consensus regarding the optimal therapeutic strategy for these patients. We report the case of a patient whose active bleeding from a periampullary duodenal diverticulum was diagnosed by side-viewing endoscopy and successfully controlled by using hemostatic forceps.

## Case presentation

A 71-year-old Asian woman with tarry stools and dyspnea was admitted to our hospital. She had a history of idiopathic interstitial pneumonia and had been taking oral prednisolone for the previous three months. She was anemic and had a pulse rate of 130 beats per minute and a systolic blood pressure of 80mmHg. Laboratory data were as follows: hemoglobin 7.2g/dL, platelet count 21.9×10^3^/mm, albumin 3.0g/dL, prothrombin time 80% and international normalized ratio 1.13, aspartate transaminase 12IU/L, alanine transaminase 17IU/L, blood urea nitrogen 32.2mg/dL, and creatinine 0.43mg/dL.

**Figure 1:**
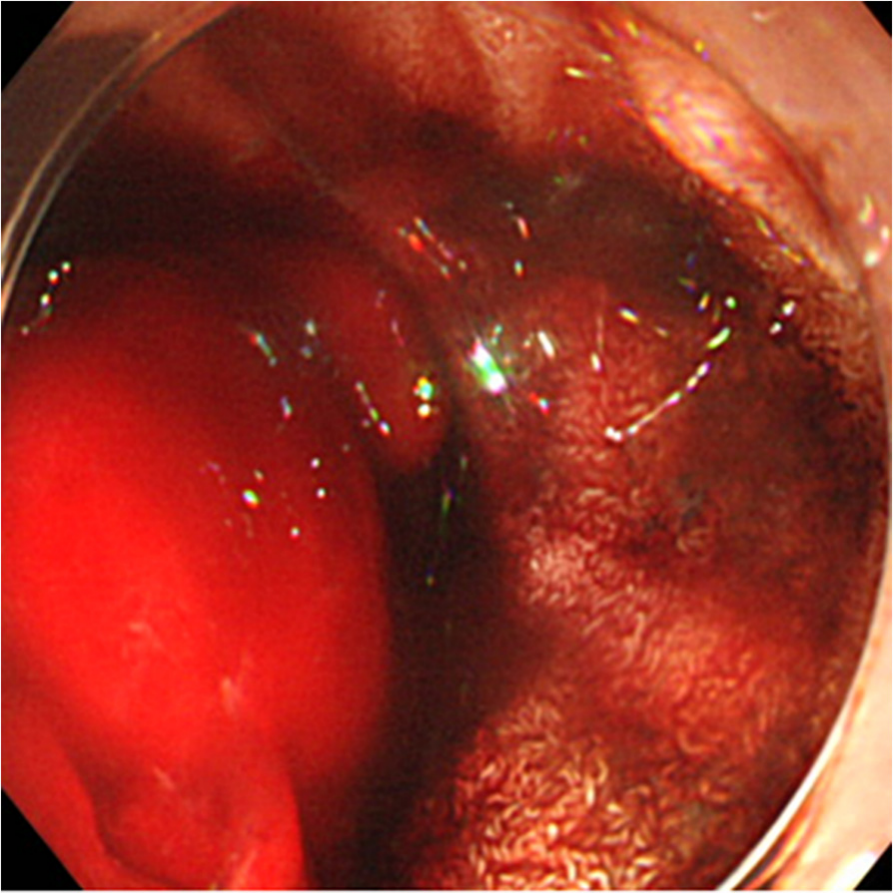
**The first endoscopic examination revealed a large clot in the duodenal bulb and fresh blood in a large periampullary diverticulum of the descending part of the duodenum.** After distilled water was flushed to remove the fresh blood, no bleeding point was identified in the periampullary diverticulum.

**Figure 2:**
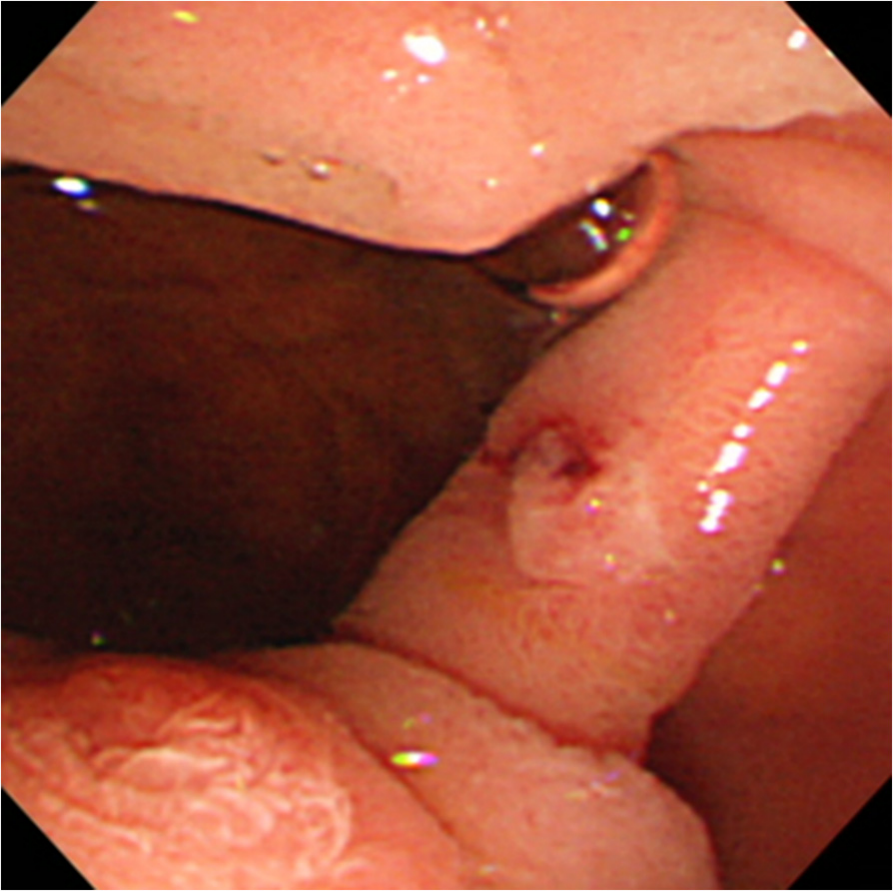
Side-viewing endoscopy revealed a small ulcer with an exposed blood vessel overlying the bile duct within the periampullary duodenal diverticulum.

**Figure 3:**
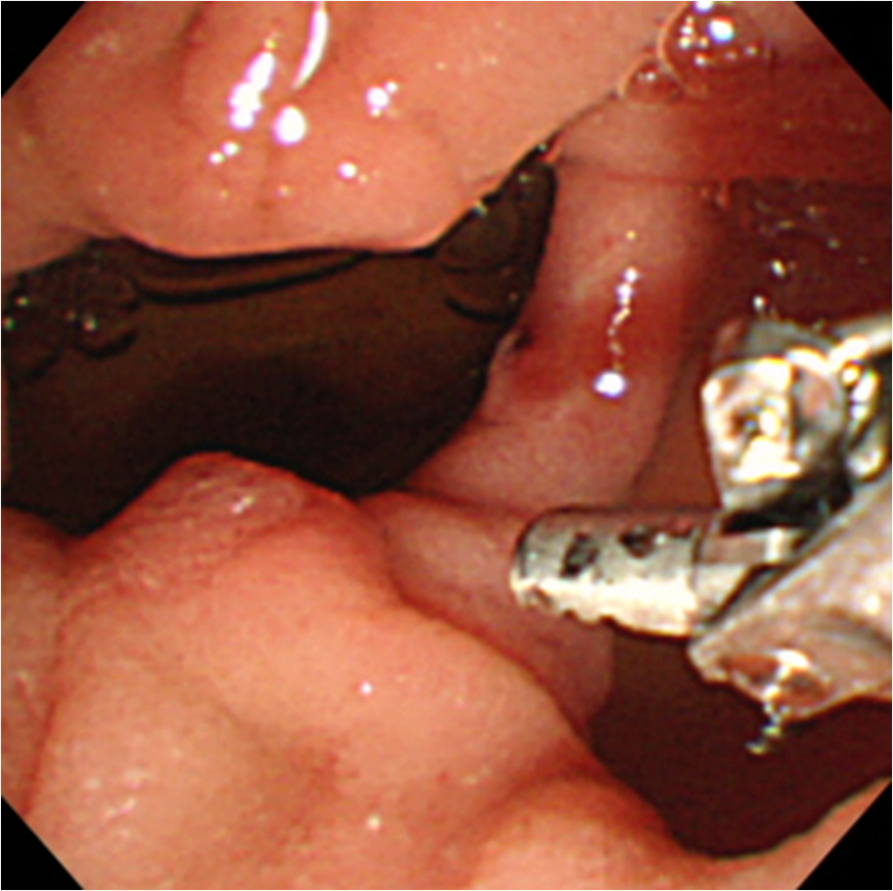
The bleeding vessel was cauterized by using hemostatic forceps in the soft coagulation mode at 80W.

**Figure 4:**
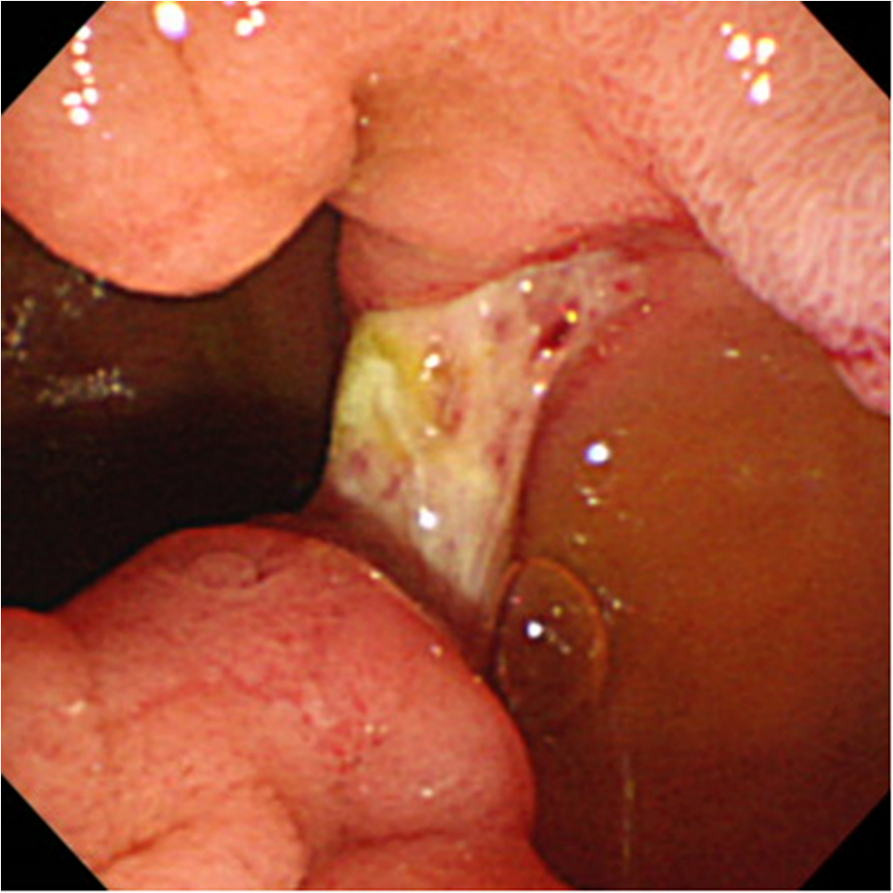
One week after cauterization, endoscopic examination showed no bleeding or exposed vessels.

After admission, we performed emergency endoscopy by using a forward-viewing endoscope (GIF-Q-260J; Olympus, Tokyo, Japan) with an attached hood (Elastic touch, slit and hole type M hood; Top Corporation., Tokyo, Japan), which showed a large clot in the duodenal bulb and a fresh clot in a large periampullary diverticulum of the descending part of the duodenum (Figure 1). However, we were unable to identify the source of the bleeding. Our patient was treated with a blood transfusion, but her severe anemia and frequent tarry stools continued. Two days later, we performed a second endoscopic examination, but this did not identify the source of the bleeding. The following day, we performed an endoscopic examination by using a side-viewing endoscope (TJF-240; Olympus), which revealed a small ulcer with a small exposed vessel overlying the bile duct within the diverticulum (Figure 2). Flushing with distilled water revealed an oozing bleeding point in the ulcer. We used hemostatic forceps (Coagrasper, FD- 410LR; Olympus) in the soft coagulation mode at 80W (VIO 300 D; ERBE,Tokyo Japan) to cauterize the bleeding vessel (Figure 3). The bleeding stopped immediately, the procedure was well tolerated by our patient, and there were no complications. A repeat examination one week later using a side-viewing endoscope showed no bleeding (Figure 4). There was no recurrence of bleeding, and our patient did not experience any complications such as perforation, cholangitis, or pancreatitis.

## Discussion

The prevalence of extragastric diverticula is increasing with the aging population. These diverticula are usually asymptomatic. Periampullary duodenal diverticula are a rare cause of gastrointestinal bleeding. A single-center clinical study found that 28% of patients undergoing endoscopic retrograde cholangiopancreatography had periampullary diverticula [5]. The most common location of duodenal diverticula is reported to be the descending part of the duodenum, accounting for 70% to 80% of cases [6]. One study reported that half of duodenal diverticula were ampullary and half were periampullary [7]. In our case, we found a small ulcer with an exposed vessel within a periampullary duodenal diverticulum. The rare location of the ulcer in the mucosa overlying the bile duct made diagnosis and treatment difficult. We were unable to identify the lesion by using forward-viewing endoscopy because of its small size and its location at about 3cm from the ampulla of Vater. We therefore performed side-viewing endoscopy to locate the lesion. A previous case report described successful diagnosis and treatment of a Dieulafoy-like lesion at the brim of a periampullary diverticulum using side-viewing endoscopy and hemoclipping [2].

In cases of diverticular bleeding, the bleeding site is usually in the dome of the diverticulum, where aberrant blood vessels lie on the thin-walled luminal surface [8]. In our case, the lesion was in the dome of the diverticulum, on an elevated area of mucosa overlying the bile duct. It is possible that this ulceration was caused by the use of steroid drugs. Endoscopy was first used by [Ryan et al.[9] and [Sim et al.[10] to identify the locations of bleeding points in duodenal diverticula. Since then, endoscopic therapy for bleeding duodenal diverticula has been reported with increasing frequency. Although various therapeutic strategies are available, we chose to use hemostatic forceps to control the bleeding because of the underlying bile duct. Deeper penetration by hemoclipping may have caused complications such as jaundice, acute cholangitis, or acute pancreatitis. We administered a protease inhibitor and an antibiotic intravenously on both the day of the procedure and the following day to prevent complications.

Using instruments through a side-viewing endoscope can be difficult because of the angle of the tip of the scope. However, therapeutic procedures can be performed by an experienced endoscopist. We used hemostatic forceps through a side-viewing endoscope to successfully control bleeding from a periampullary diverticulum without complications.

## Conclusions

Bleeding from a duodenal diverticulum may be fatal. Recently, capsule endoscopy has been used with increasing frequency to diagnose obscure gastrointestinal bleeding. Rapid diagnosis and treatment of duodenal diverticula are often difficult using forward-viewing endoscopy, and another instrument such as a side-viewing endoscope may be needed. If the source of bleeding is not identified during the first endoscopic examination, repeat examination is necessary. Hemostatic forceps are approved for the treatment of bleeding from both gastric ulcers and duodenal diverticula. Use of hemostatic forceps to control bleeding from an ulcer overlying the bile duct decreases the risk of acute cholangitis or pancreatitis. We present a rare case in which hemostatic forceps were used to treat a bleeding point located in a duodenal diverticulum.

## Consent

Written informed consent was obtained from the patient for publication of this manuscript and any accompanying images. A copy of the written consent is available for review by the Editor-in-Chief of this journal.

## Competing interests

The authors declare that they have no competing interests.

## Authors’ contributions

All of the authors contributed significantly to the manuscript and read and approved the final version. NN coordinated the writing of the manuscript and wrote the initial draft. HM, HK, SF, MK, and TM researched the literature. KR critically revised the manuscript.
